# Ultrasound-guided percutaneous injection of *Pseudomonas aeruginosa*-mannose sensitive hemagglutinin for treatment of chyle fistula following neck dissection: Two case reports

**DOI:** 10.1097/MD.0000000000018816

**Published:** 2020-01-31

**Authors:** Qiang Chen, Yanling Chen, Anping Su, Yu Ma, Boyang Yu, Xiuhe Zou, Dongmei Peng, Jingqiang Zhu

**Affiliations:** a Department of Thyroid and Parathyroid Surgery Center; b Department of Rheumatology and Immunology; c Department of Ultrasonography, West China Hospital, Sichuan University, Chengdu, China.

**Keywords:** chyle fistula, neck dissection, *Pseudomonas aeruginosa*-mannose sensitive hemagglutinin, sclerotherapy, ultrasonography

## Abstract

**Rationale:**

Chyle fistula is a rare but troublesome complication of neck dissection. Topical application of *Pseudomonas aeruginosa*-mannose sensitive hemagglutinin (PA-MSHA) injection has been reported as a novel, viable, and effective approach in the treatment of chyle fistula following neck dissection. However, there have been no reports regarding the treatment of chyle fistula using ultrasound (US)-guided percutaneous injection of PA-MSHA.

**Patient concerns:**

We describe 2 patients with thyroid cancer who developed chyle fistula following neck dissection, which remained unresolved despite the use of conservative treatment.

**Diagnoses:**

Both the patients were diagnosed with chyle fistula by laboratory testing, which showed that drainage fluid triglyceride concentration was >100 mg/dL.

**Interventions:**

When conservative treatment failed, a 2 mL undiluted PA-MSHA preparation was percutaneously injected at the effusion site of the left supraclavicular area under US guidance with aseptic technique. Concomitantly, the drainage tube was clamped for at least 30 minutes.

**Outcomes:**

Chyle fistula in both patients were successfully resolved with this technique within 2 or 4 days, without notable side effects.

**Lessons:**

US-guided percutaneous injection of PA-MSHA is a simple and effective method to treat chyle fistula following neck dissection, which may serve as a useful addition to the medical treatment for cervical chyle fistula.

## 1 Introduction

Chyle fistula is defined as a leakage of lymphatic fluid from the thoracic duct or lymphatic vessels, which typically manifests as increased drainage of a milky or ivory white fluid. In the literature, the incidence of chyle fistula following neck dissection ranges from 0.5% to 8.3%.^[1–4]^ It is usually associated with prolonged hospitalization and may lead to metabolic imbalances, nutritional deficiencies, and immunological dysfunctions owing to the persistent loss of protein and electrolyte-rich fluid.^[5–8]^ Although many medical methods have been adopted to treat cervical chyle fistula, an optimal treatment regimen has not yet been determined. Most instances of chyle fistulae following neck dissection can be resolved successfully through conservative treatment. Diet modification, local compressive dressing, negative-pressure drainage, and octreotide therapy are the main approaches for initial treatment of chyle fistula.^[5–7,9]^ However, conservative treatment is reportedly ineffective for some patients; in such situations, surgical intervention has been necessary. Surgical intervention for chyle fistula has traditionally consisted of re-exploration. However, some patients may be unable to tolerate an early reoperation. Additionally, identification of a diffuse leakage site in the secondary surgery area is difficult and potentially morbid, especially when performed by an inexperienced surgeon.^[10]^ Hence, it is necessary to explore less invasive surgical approaches and nonsurgical therapies.

**Figure 1:**
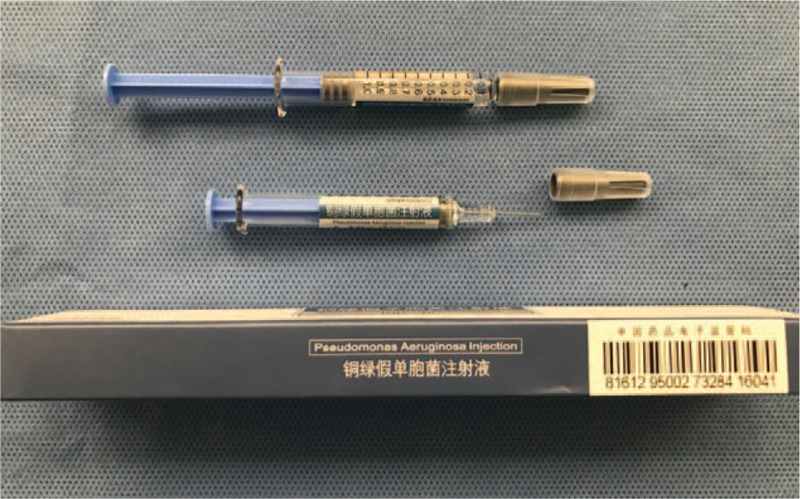
*P aeruginosa*-mannose sensitive hemagglutinin injection and its outer packaging.

Topical injection of *Pseudomonas aeruginosa*-mannose sensitive hemagglutinin (PA-MSHA, Beijing Wanter Bio-pharmaceutical Company, Beijing, China) (Fig. 1) has been suggested as a feasible treatment for chyle fistula following neck dissection; to date, it has shown no considerable side effects.^[11]^ The proposed mechanism by which PA-MSHA can treat chyle fistula is similar to that of other sclerosing agents: it initiates of a local strong inflammatory response between tissue surfaces, which results in fibrotic adhesion and spontaneous fistula closure.^[11]^

With the development of high-resolution ultrasound (US), high-precision injection and aspiration therapies have become possible. US-guided sclerotherapy is a minimally invasive approach that has been widely used to treat lymphatic or venous malformations of the head and neck.^[12,13]^ To the best of our knowledge, there has been no report regarding the treatment of chyle fistula using US-guided percutaneous injection with PA-MSHA. Here, we describe 2 patients with chyle fistula following neck dissection who failed in conservative treatment but were successfully resolved by percutaneous injection of PA-MSHA under US guidance.

## 2 Case reports

### 2.1 Case 1

A 30-year-old man presented with a 4-month history of a thyroid mass. Physical examination revealed a 1 × 1-cm, indurated and poorly demarcated mass in the left lobe. US showed an inhomogeneous hypoechoic nodule with unclear margin and microcalcification in the upper portion of the left thyroid lobe, along with multiple suspicious enlarged lymph nodes in the left lateral neck. The patient was diagnosed with papillary thyroid carcinoma with cervical lymph node metastasis by preoperative fine-needle aspiration biopsy. Subsequently, a total thyroidectomy, central and left modified radical lateral neck dissection was performed. Chyle leakage appeared during removal of the lymph nodes at level IV. With meticulous ligation of the thoracic duct and lymphatic vessels, the chyle fistula was controlled. No obvious chyle leakage was observed at the end of the operation. Two closed suction drains were placed in the central and left neck, respectively.

**Figure 2:**
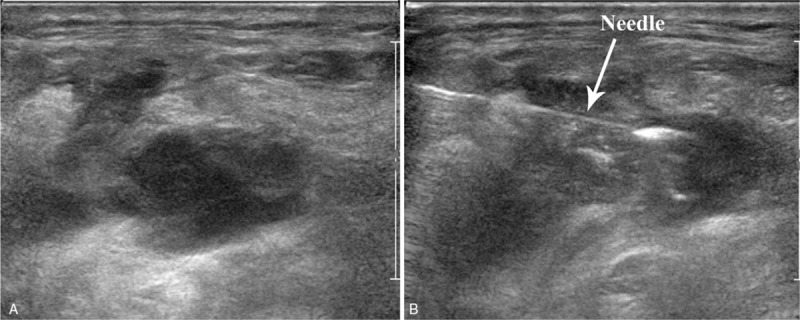
(A) Transverse ultrasound view of the left supraclavicular area shows the location of the effusion site. (B) Ultrasound image shows needle placement (arrow) at the effusion site during real-time ultrasound-guided injection of *P aeruginosa*-mannose sensitive hemagglutinin preparation.

On postoperative day (POD) 2, the patient's 24-hour drainage volume in the left neck increased to 475 mL with a whitish milky appearance after oral feeding. Postoperative chyle fistula was suspected, and the drainage fluid was sent for biochemical analysis. The result showed a triglyceride level of 459 mg/dL, which was indicative of chylous fluid. The patient was started on a fat-free diet and received negative-pressure drainage. Three days later, the drainage output showed a declining tendency, but remained >100 mL/d. On POD 7, a total of 2 mL undiluted PA-MSHA preparation (total bacterial count: 1.0 × 10 8 cfu/mL) was injected at the effusion site of the left supraclavicular area under US guidance with aseptic technique (Fig. 2A and B). The chyle drainage output rapidly declined within 12 hours after the injection and ceased on the following day. On POD 9, a regular diet was given, and on POD 10, the patient was discharged after removal of the drains. At the 6-month of follow-up, there was no evidence of neck effusion, seroma, wound infection, or other complications.

### 2.2 Case 2

A 44-year-old woman was referred to our hospital with consideration of recurrent thyroid cancer. Two years prior to admission, she had undergone a total thyroidectomy and central neck dissection and had received 100 mCi of radioactive iodine in an outside hospital. US showed multiple enlarged lymph nodes with microcalcification in the lower internal jugular vein (IJV) of the left neck. Fine-needle aspiration biopsy of the lymph nodes confirmed the diagnosis of recurrent papillary thyroid carcinoma. Contrast-enhanced computed tomography scan showed no obvious abnormalities and no space-occupying lesions in the lungs. A left modified radical neck dissection was performed subsequently. During neck dissection, chyle leakage was identified in the left supraclavicular region after resection of the lymph nodes around the junction of the IJV and subclavian vein. Chyle leakage was controlled by ligation of the thoracic duct and its multiple branches. However, it was observed again prior to skin closure; therefore, the leakage site was oversewn with a nonabsorbable suture (5/0 Prolene). A closed suction drain was placed in the left supraclavicular fossa.

**Figure 3:**
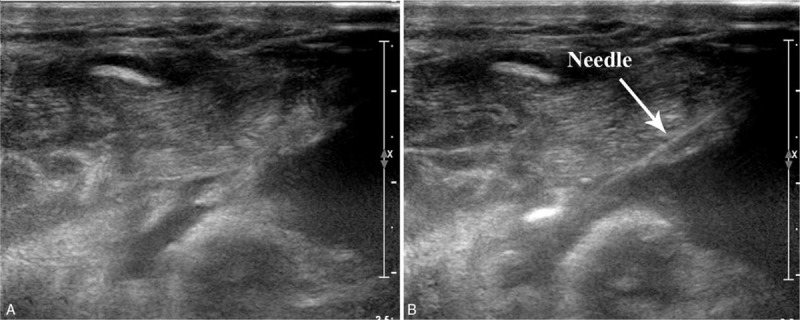
(A) Transverse ultrasound view of the left supraclavicular area shows the location of the effusion site. (B) Ultrasound image confirms needle placement (arrow) at the effusion site during real-time ultrasound-guided injection of *P aeruginosa*-mannose sensitive hemagglutinin preparation.

On POD 1, 150 mL of serosanguineous fluid was drained and the patient was prescribed an oral light diet. However, a total of 785 mL of slightly milky fluid appeared in the drain on POD 2. A sample of the fluid was sent for laboratory testing, which revealed that it was chylous fluid with an elevated triglyceride level of 726 mg/dL. An oral diet consisting of medium-chain triglycerides (MCTs) was initiated, in combination with the application of compressive dressing and negative-pressure drainage. On POD 4, the 24-hour neck drainage volume was 1470 mL. Oral diet was discontinued and total parenteral nutrition (TPN) was commenced. In addition, subcutaneous octreotide therapy (Novartis Pharma Schweiz AG, Basel, Switzerland) was initiated at a dose of 100 μg/8 h. Daily drainage volume showed a considerable reduction with these managements, but remained approximately 300 to 400 mL/d over the following 3 days. On POD 8, the daily volume of drainage volume remained >300 mL/d; thus, a total of 2 mL undiluted PA-MSHA preparation (total bacterial count: 1.0 × 10 8 cfu/mL) was injected at the effusion site of the left supraclavicular area under US guidance (Fig. 3A and B). Drainage decreased to 100 mL/d over the next 2 days; it was <20 mL on POD 11. A mild-grade fever was the only adverse event associated with this treatment, and was controlled by administration of nonsteroidal anti-inflammatory drugs. Enteral feeding was resumed on POD 12 without the recurrence of leakage. On POD 14, the closed suction drain was removed and the patient was discharged. The patient was followed-up for 6 months, during which no neck swelling, seroma, or wound infection developed.

This study was approved by the Institutional Review Board of West China Hospital, Sichuan University, and the patients and their family have provided written informed consent for publication of the case reports.

### 2.3 US-guided injection technique

US-guided percutaneous injection of PA-MSHA was performed after informed consent was obtained and puncture-related contraindications were excluded. The patient was placed in the supine position and a pillow was placed behind the patient's back to maintain head-back position. Prior to the injection, conventional US was performed to identify the effusion site and adjacent anatomic structures (artery, vein, and surrounding important structures). After determining the puncture site, the skin of the puncture site was cleaned and disinfected (1% chlorhexidine alcohol solution), then covered with a sterile drape. The US transducer was also covered with a sterile sheath. After subcutaneous infiltration of local anesthetic (1% lidocaine), a 25-gauge needle was used to inject 2 mL PA-MSHA preparation at the effusion site of the left supraclavicular area under real-time US guidance. The puncture site was pressed directly with sterile dressing for 5 minutes to prevent bleeding or hematoma after withdrawal of the needle.

## 3 Discussion

Chyle fistula is a rare complication of neck dissection, which can be life-threatening in some patients. Treatment of postoperative chyle fistula is unsatisfactory and often difficult and there are no standardized guidelines for such treatment. Meticulous identification and ligation of the thoracic duct and multiple variant lymph channels during the neck dissection is the key step to prevent postoperative chyle fistula.^[2,8,10]^ Intraoperative chyle fistula is occasionally encountered during lateral neck dissection and can be controlled through careful ligation or suture ligation of the thoracic duct or lymph vessels in most patients.^[2]^ Despite these preventive measures, postoperative chyle leakage develops in some patients.

Management of postoperative chyle leakage includes conservative management and surgical intervention. Conservative management is widely regarded as the first-line approach for treatment of postoperative chyle leakage. The fundamental aspects of conservative treatment include bed rest, local compressive dressing, negative-pressure drainage, and nutritional modifications, including a fat-free diet, a MCTs diet or TPN.^[14]^ The main goal of nutritional modification is promotion of spontaneous fistula closure by diminishing chyle production and flow.^[6,9,15]^ The application of somatostatin and its long-acting synthetic analogue, octreotide, in the treatment of chyle fistula is a novel, feasible, and effective approach.^[5–7]^ Octreotide can inhabit secretion of multiple hormones; however, the probable mechanism by which it promotes chyle leakage closure is related to its action on decreasing blood flow to the hepatic, portal, and splanchnic circulation.^[5–7,9]^

PA-MSHA was initially used for the treatment of recurrent axillary seroma and has reportedly been effective in treating malignant pleural effusion and pericardial effusion.^[16,17]^ Successful management of refractory chyle fistula by topical injection of PA-MSHA via a drainage tube was first reported by Wei et al in 2015.^[11]^ PA-MSHA is a type of genetically engineered *P aeruginosa*, in which mycelium is covered with many tenuous and upright MSHA fimbriae.^[18,19]^ Notably, these fimbriae can activate Th1-type immune responses, thereby promoting the maturation and migration of natural killer cells, macrophages, and dendritic cells.^[18–20]^ Given the anti-tumor effect of PA-MSHA, it has been approved by the China Food and Drug Administration and can serve as an adjuvant therapy for malignant tumors.^[21–23]^

US-guided injection of drugs is an easily accessible and minimally invasive technique that has become a useful tool in rehabilitation medicine; it is widely used to treat subacromial bursitis, carpal tunnel syndrome, and De Quervain tendinopathy.^[24–27]^ US-guided sclerotherapy is reported to be a safe, effective, and inexpensive procedure for treating benign cystic masses in the neck.^[13]^ Herein, we have reported the first 2 patients in which chyle fistula was successfully resolved by US-guided percutaneous injection of PA-MSHA.

In this report, 2 patients with postoperative chyle fistula that did not resolve with the conservative treatment mentioned above; however, both achieved resolution within 2 or 4 days after PA-MSHA treatment, without notable side effects. These findings suggest that US-guided percutaneous injection of PA-MSHA is safe and effective for the treatment of chyle fistula. Compared with blind injection, the advantages of US-guided injection are obvious. The needle can be targeted accurately with US guidance and the entire puncture process can be monitored in real time, which reduces the risk of damage to nerves, vessels, and other important structures.^[26,27]^ Injection of PA-MSHA through a drainage tube to treat chyle fistula following neck dissection, as reported by Wei et al,^[11]^ is also a viable and effective method; however, it requires an additional 5 mL of saline to flush the lumen to ensure that the drug can reach the surgical area. However, this approach inevitably causes dilution of the drug, which may lower the therapeutic effects of PA-MSHA. In addition, whether PA-MSHA can accurately reach the leakage site via drainage tube injection is closely related to the placement of the drainage tube and the position of the patient. In contrast, undiluted PA-MSHA can be injected directly and accurately at the leakage site under US guidance.

If the chyle fistula remained and showed no sign of resolution after the injection of PA-MSHA, surgical intervention would be necessary. Surgical re-exploration has traditionally been implemented as the standard treatment for chyle fistula, when conservative management is unsuccessful.^[2,9,10]^ In previous reports, some investigators have noted that sclerotherapy may increase the difficulty of subsequent surgical re-exploration; thus, sclerotherapy should be performed cautiously.^[28]^ However, it is unlikely that US-guided PA-MSHA injection would considerably complicate surgical re-exploration because the injection involves a small volume that is precisely delivered to the effusion site with US guidance.

In conclusion, US-guided percutaneous injection of PA-MSHA is a simple and effective method to treat chyle fistula following neck dissection. The rapid response and minimal side effect profile may make this therapeutic approach a promising addition to the medical treatment available for patients with cervical chyle fistula. However, additional studies are needed to evaluate the safety and efficacy of US-guided percutaneous injection of PA-MSHA for the treatment of chyle fistula.

## Author contributions

**Conceptualization:** Qiang Chen, Anping Su, Yu Ma, Boyang Yu, Xiuhe Zou, Jingqiang Zhu.

**Methodology:** Anping Su, Yu Ma, Xiuhe Zou, Jingqiang Zhu.

**Visualization:** Yanling Chen, Boyang Yu, Dongmei Peng.

**Writing – original draft:** Qiang Chen, Yanling Chen, Anping Su.

**Writing – review & editing:** Jingqiang Zhu.
